# Extremely low lattice thermal conductivity in light-element solid materials

**DOI:** 10.1093/nsr/nwae345

**Published:** 2024-09-28

**Authors:** Ni Ma, Lu Liu, Runhua Wu, Juping Xu, Wen Yin, Kai Li, Wei Bai, Jiong Yang, Chong Xiao, Yi Xie

**Affiliations:** Hefei National Laboratory for Physical Sciences at the Microscale, University of Science and Technology of China, Hefei 230026, China; Materials Genome Institute, Shanghai University, Shanghai 200444, China; Hefei National Laboratory for Physical Sciences at the Microscale, University of Science and Technology of China, Hefei 230026, China; Spallation Neutron Source Science Center, Dongguan 523803, China; Spallation Neutron Source Science Center, Dongguan 523803, China; Hefei National Laboratory for Physical Sciences at the Microscale, University of Science and Technology of China, Hefei 230026, China; Hefei National Laboratory for Physical Sciences at the Microscale, University of Science and Technology of China, Hefei 230026, China; Materials Genome Institute, Shanghai University, Shanghai 200444, China; Zhejiang Laboratory, Hangzhou 311100, China; Hefei National Laboratory for Physical Sciences at the Microscale, University of Science and Technology of China, Hefei 230026, China; Hefei National Laboratory for Physical Sciences at the Microscale, University of Science and Technology of China, Hefei 230026, China

**Keywords:** low lattice thermal conductivity, light-element materials, square-net lattice

## Abstract

Lattice thermal conductivity (*κ*_l_) is of great importance in basic sciences and in energy conversion applications. However, low-*κ*_l_ crystalline materials have only been obtained from heavy elements, which typically exhibit poor stability and possible toxicity. Thus, low-*κ*_l_ materials composed of light elements should be explored. Herein, light elements with hierarchical structures in a simple square-net lattice as well as a small discrepancy in atomic mass and radius exhibit low *κ*_l_. The hierarchical structure exhibits various chemical bonds and asymmetric geometry of building units, resulting in flat phonon branches and strong phonon–phonon interactions similar to those observed in heavy-element materials. These phenomena generate a large phonon anharmonicity, which is the prerequisite for achieving extremely low *κ*_l_. For example, KCu_4_Se_3_ exhibits an extremely low *κ*_l_ of 0.12 W/(m·K) at 573 K, which is lower than that of most heavy-element materials. These findings can reshape our fundamental understanding of thermal transport properties of materials and advance the design of low-*κ*_l_ solids comprising light elements.

## Teaser

This work demonstrates two square-net chalcogenides, composed of relatively light elements with small atomic numbers, that exhibit extremely low lattice thermal conductivity.

## INTRODUCTION

Lattice thermal conductivity (*κ*_l_) is a fundamental and inherent physical property of all solids that characterizes the transport properties of heat-carrying phonons. Lattice thermal conductivity is an important parameter in basic science, such as solid chemistry and condensed matter physics [1,2], as well as in energy conversion applications such as thermoelectrics [3], thermal insulation [4] and thermal barrier coatings [5]. Since Einstein published his theoretical work [6], achieving low *κ*_l_ and understanding the underlying physics of thermal transport became the main requirements for these thermal applications. However, these requirements still represent a challenge because of the difficulty of modeling the complex many-body interactions in solids [1,2]. Thus, developing new materials with low intrinsic *κ*_l_ is essential from a scientific and technological perspective.

The current research indicates that low *κ*_l_ can be achieved in solids with large-atomic-mass elements such as PbTe, which consists of heavy elements [7,8]; materials with heavy elements that exhibit rattling vibration modes, such as Tl_3_VSe_4_ and TlInTe_2_ [9–12]; and solids with complex unit cells, such as clathrates and skutterudites [13–22]. These materials exhibit weak chemical bonding, which causes strong lattice anharmonicity, leading to a low intrinsic *κ*_l_. However, heavy atoms, such as those of Pb and Tl, are typically accompanied by weak chemical bonding and toxicity, resulting in poor stability and environmental pollution. Thus, it is crucial to broaden the range of materials with low *κ*_l_ by thoroughly exploring the low intrinsic *κ*_l_ in new solids consisting of earth-abundant and non-toxic light elements. This may offer new atomistic insights into the physical origins and dynamics of phonon scattering.

To achieve low *κ*_l_ in solids composed of light elements, i.e. with low atomic mass, the square-net lattice, which is the simplest fundamental lattice topology, is employed because it is relatively easy to theoretically model. This lattice type exhibits remarkably abundant atomic compositions as well as electronic and crystal structure diversities [23]. Moreover, the distances among the neighboring atoms and between the interlayers in square-net lattices [24] provide them with a chemical bond hierarchy and various vibration modes, causing large anharmonicity similar to that in heavy-element materials. Thus, square-net lattices can be a structural motif that acts as a platform for large phonon interactions, resulting in low *κ*_l_.

In this study, square-net KCu_4_S_3_ and KCu_4_Se_3_ were studied as examples to explore the low *κ*_l_ in solids with light elements. These simple solids are light and exhibit low intrinsic *κ*_l_. The small discrepancy in mass and small radius contrast ratio along with the structural hierarchy of these solids contribute to their strong phonon–phonon interactions, resulting in flat phonon branches and large phonon anharmonicity, which was confirmed by the large Grüneisen parameter and numerous scattering channels with a large three-phonon phase space. As a result, KCu_4_S_3_ and KCu_4_Se_3_ show low *κ*_l_ of 0.15 and 0.12 W/(m·K), respectively, at 573 K. The results provide new insights into the phonon transport in light elements and contribute to the search for new low-*κ*_l_ solids comprising light and earth-abundant elements with low toxicity.

## RESULTS AND DISCUSSION

**Figure 1.**
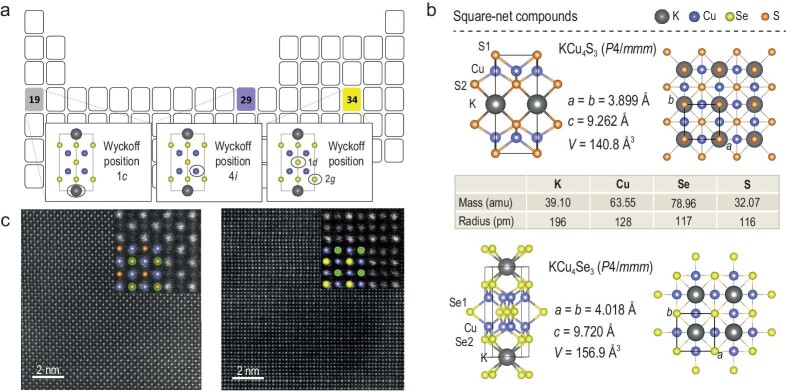
(a) Schematic of the periodic table, with potassium (K), copper (Cu) and selenium (Se) highlighted in gray, purple and yellow, respectively. (b) Side and top structural views of KCu_4_S_3_ and KCu_4_ Se_3_ with their lattice parameters marked. (The atomic masses and atomic radii of these light elements are listed in Table). (c) The high-angle annular dark-field (HAADF) images of KCu_4_S_3_ (left) and KCu_4_Se_3_ (right) with the (00*l*) orientation, showing the square-net topology. Their projection structures are separately given in the inset. (Color codes: green, blue, orange and yellow for K, Cu, S and Se, respectively).

KCu_4_S_3_ and KCu_4_Se_3_ are isostructural compounds with small lattice parameters, and they crystallize in the *P*4/*mmm* space group. The light elements in KCu_4_Se_3_ are highlighted in the periodic table in Fig. 1a with K, Cu and Se marked in gray, blue and yellow, respectively. As shown in Fig. 1b, the lattice parameters of KCu_4_S_3_ are *a* = *b* = 3.899 Å and *c* = 9.262 Å, while *V* = 140.8 Å^3^, and those of KCu_4_Se_3_ are *a* = *b* = 4.018 Å and *c* = 9.720 Å, while *V* = 156.9 Å^3^. Moreover, the atomic mass and atomic radius of K, Cu, Se and S are 39.1 amu and 196 pm, 63.55 amu and 128 pm, 78.96 amu and 117 pm, and 32.07 amu and 116 pm, respectively. The mass contrast ratio and discrepancy in atomic radius among these elements are small. Moreover, their structures consist of infinite [(Cu^+^)_4_(Se^2−^)_2_](Se^−^) or [(Cu^+^)_4_(S^2−^)_2_](S^−^) layers with their charge balanced by ionic K^+^. Each atomic layer shows distinctive square-net topology along the 001 direction, with the nearest atomic distance of K–K in KCu_4_S_3_ being *d*_K–K_ = *a* = 3.899 Å, and of K–K or Se–Se in KCu_4_Se_3_ being *d*_K–K_ or *d*_Se–Se_ = *a* = 4.018 Å; the nearest atomic Cu–Cu distance in KCu_4_S_3_ and KCu_4_Se_3_ is *d*_Cu–Cu_ = √2/2*a* = 2.757 and 2.841 Å, respectively. Lamellar crystals of KCu_4_S_3_ and KCu_4_Se_3_ were prepared using the boron–chalcogen method (refer to the supplementary data for more details) [19], see scanning electron microscopy images (Table S1), the semiquantitative elemental analysis was conducted using energy-dispersive spectrometry, which indicated that the average formulas of the two compounds are KCu_4.0(7)_S_3.0(5)_ and KCu_4.2(1)_Se_3.0(1)_. In addition, high-angle annular dark-field scanning transmission electron microscopy (HAADF–STEM) was performed to investigate the square-net topology of these compounds along the 001 direction. The top (00*l*) structure view of these two compounds was readily obtained owing to their layered structure (Fig. 1c). Both KCu_4_S_3_ and KCu_4_Se_3_ exhibit a square-net topology. For clarity, the expanded view of the HAADF–STEM image of KCu_4_S_3_ (left panel) and KCu_4_Se_3_ (right panel) are given along with their projection structure (the color code was set as green for K, blue for Cu, orange for S, and yellow for Se). The atoms were distinguished via mass contrast. In the case of KCu_4_S_3_, the bright spots reflect Cu atoms (atomic mass = 63.55 amu, nearest *d*_Cu–Cu_ ≈ 2.738 Å), whereas the dark spots reflect the S atoms (atomic mass = 32.07 amu), some of which overlap with K atoms. In KCu_4_Se_3_, the bright spots reflect Se atoms, with some Se atoms overlapping with K, and the dark spots reflect Cu atoms, with the nearest *d*_Cu–Cu_ ≈ 2.864 Å, which is in good agreement with the theoretical crystal structure.

**Figure 2.**
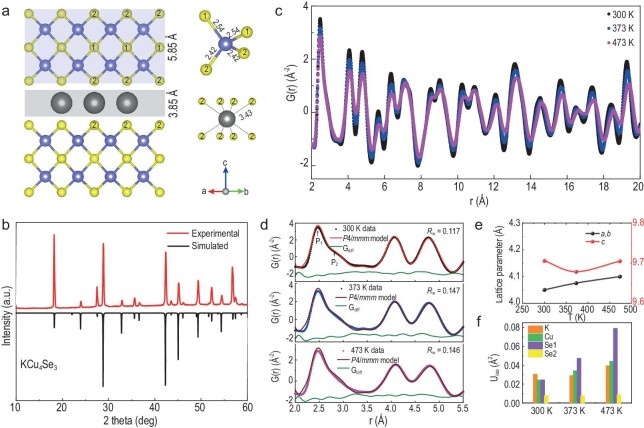
(a) (110) structure view of KCu_4_Se_3_ (blue shading: [(Cu^+^)_4_(Se^2−^)_2_](Se^−^) and gray shading: [(K^+^)(Se^2−^)]^−^ sublattice), and the local coordination environments of Cu and K. (b) Powder X-ray diffraction (PXRD) patterns of KCu_4_Se_3_. (c) Neutron atomic pair distribution function (PDF) data (*r* = 2–20 Å) of KCu_4_Se_3_ at 300 K (black), 373 K (blue) and 473 K (pink). (d) Neutron PDF fitting of the local structure (*r* = 2–5.5 Å) of KCu_4_Se_3_ at 300 K, 373 K and 473 K. (e) Temperature-dependent lattice parameter and (f) isotropic atomic displacement parameters (U_iso_) of KCu_4_Se_3_ based on the *P*4/*mmm* model fitting of the PDF data.

The crystal structure of KCu_4_Se_3_ was studied as an example [25] to understand the structure of these materials. Figure 2a depicts the structure view of the (110) plane, showing the [(Cu^+^)_4_(Se^2−^)_2_](Se^−^) sublattice with an interlayer distance (*d*_Cu–Se layer_) of ∼5.85 Å and the [(K^+^)(Se^2−^)]^−^ sublattice with a *d*_K–Se layer_ of 3.85 Å, confirming the bonding hierarchy in KCu_4_Se_3_. The unit cell is composed of four crystallographically independent atoms: K (1*c* Wyckoff site), Cu atom (4*i* Wyckoff site), Se1 (1*d* Wyckoff site) and Se2 (2*d* Wyckoff site). The Cu atoms adopt a distorted CuSe_4_ tetrahedra with two Cu–Se1 and two Cu–Se2 bonds, whereas K is surrounded by eight Se1 atoms (right panel of Fig. 2a). Cu and K exhibit different local chemical environments, with their bond distances in the following order: K–Se2 (3.43 Å) > Cu–Se1 (2.54 Å) > Cu–Se2 (2.42 Å), suggesting their crystallographically different bonding strength. Moreover, the valence states of K, Cu, Se1 and Se2 were determined, using X-ray photoelectron spectroscopy, to be 1+, 1+, 1− and 2−, respectively (Fig. S2). The monochalcogenides with −1 charge are delocalized as holes, resulting in a weak Cu–Se1(Se^−^) chemical bonding with a longer bond length. Similar bonding is observed in related copper chalcogenides such as the isostructural CsCu_4_Se_3_ [19]. These results indicate that the three clear characteristics of KCu_4_S_3_ and KCu_4_Se_3_ are (i) a simple square-net lattice with small lattice parameters, (ii) light-element constituents with small mass and radius contrast ratio, and (iii) tetragonal structures with distinct bonding hierarchy along the crystallographic *c*-axis.

Herein, we successfully synthesized the single phase of these two compounds, and their powder X-ray diffraction (PXRD) patterns are shown in Fig. S1. The PXRD pattern of KCu_4_Se_3_ (Fig. 2b) can be attributed to the tetragonal *P*4/*mmm* space group. In addition, the neutron atomic pair distribution function (PDF) was used to investigate the local interatomic structure of KCu_4_Se_3_, which is obtained based on the Fourier transform of the total neutron scattering data. G(*r*) is the Fourier transform of the experimentally scattering structure function (S(*Q*), refer to Fig. S3 for more details). Figure 2c shows the PDF data of KCu_4_Se_3_ at an atomic distance up to 20 Å at 300 K, 373 K and 473 K. The average structure can be well explained based on its tetragonal model (*P*4/*mmm*) at a goodness of fit value *R*_w_ lower than 0.16 (refer to Fig. S4 for more details). Furthermore, Fig. 2d shows the experimental and fitted G(*r*) of KCu_4_Se_3_ up to 5.5 Å at 300 K (black line), 373 K (blue line) and 473 K (pink line), indicating the good agreement between the measured and calculated PDF curves. The first peak (P_1_) observed at 2.48 Å corresponds to the Cu–Se distance, whereas P_2_, which was observed at 2.84 Å, corresponds to the Cu–Cu distance and it has a considerably smaller magnitude. The clear asymmetry in the first peak (P_1_) can be attributed to the intrinsic distorted CuSe_4_ tetrahedra, in which there are different Cu–Se distances (Cu–Se1 vs. Cu–Se2: 2.54 Å vs. 2.42 Å) and Se2–Cu–Se2 shows a larger bond angle (111.7°) than that of Se1–Cu–Se1 (104.2°) owing to the larger space in the [(K^+^)(Se2^2−^)]^−^ interlayer (Fig. 2a). Figure 2e and f show the lattice parameters and isotropic atomic displacement parameters (U_iso_) via the tetragonal fitting within 2–5.5 Å. The lattice parameters (*a* and *b*) increase with the increase in temperature, whereas the *c* lattice parameter shows slightly different trends in Fig. 2e. This anomalous behavior in KCu_4_Se_3_ may be closely related to the atomic displacement parameters of K (Fig. 2f), and the U_iso_ of K at 373 K is lower than that at 300 K. Moreover, Cu and Se1 show large U_iso_ with an increase in temperature, suggesting that the weak Cu–Se1(Se^−^) bonding may affect the thermal transport properties of KCu_4_Se_3_.

**Figure 3.**
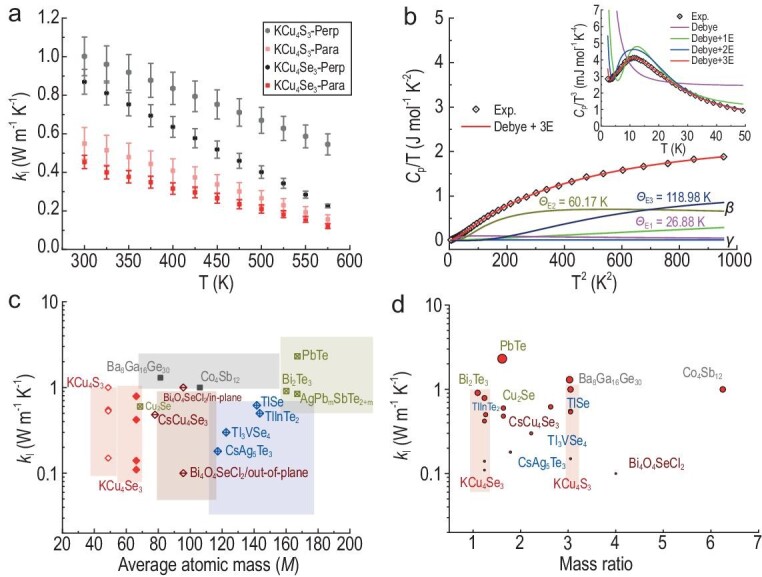
(a) Experimental low lattice thermal conductivity (*κ*_l_) of KCu_4_S_3_ and KCu_4_Se_3_ with labeled error bars. (Perp and Para are the perpendicular and parallel directions, respectively, to the SPS pressure.) (b) *C* _p_/*T* vs. *T*^ 2^ of KCu_ 4_Se_ 3_ fitted using a combination of Debye+3 Einstein models. Inset: *C*_p_/*T*^3^ vs. *T* plots to highlight the Einstein contributions (Debye model in pink, Debye+1 Einstein model in green, Debye+2 Einstein model in blue, Debye+3 Einstein model in red.) (c) Map of the experimental *κ*_l_ values of the bulk materials with different average atomic mass (*M*). KCu_4_S_3_ and KCu_4_Se_3_ exhibit small *M* and low *κ*_l_ and are highlighted by red shading. (d) *κ*_l_ vs. the mass contrast ratio of the constituent elements.

The thermal transport properties of these two compounds were then studied. First, the finely ground samples were densified into a pellet via spark plasma sintering (SPS). The densified pellets were cut along directions perpendicular (perp) and parallel (para) to the SPS pressure and were then used to measure the transport properties. The total thermal and electrical transport properties of KCu_4_S_3_ and KCu_4_Se_3_ indicate the presence of anisotropy along the pressure direction (for more details, refer to Figs S5 and S6). The intrinsically low *κ*_l_ was obtained by subtracting the *κ*_ele_ component obtained from the Wiedemann–Franz law [26], *κ*_ele_ = *LσT*, where *L, σ* and *T* are the Lorenz number, electrical conductivity and temperature, respectively. As shown in Fig. 3a, the *κ*_l_ values of KCu_4_S_3_ are 0.54–0.15 and 1.0–0.54 W/(m·K) along the para and perp directions to the SPS pressure, respectively, whereas those of KCu_4_Se_3_ are 0.43–0.12 and 0.86–0.21 in the para and perp directions, respectively, at 300 K–573 K. In contrast to the common criteria for low intrinsic *κ*_l_ in solids, which involves the presence of heavy elements or large complex unit cells [2,3], KCu_4_S_3_ and KCu_4_Se_3_ contain light-element constituents and simple unit cells involving small volume (*V*) and a small number of atoms per formula unit (*N* = 8). Therefore, achieving low *κ*_l_ in these materials is considered a breakthrough. In addition, the heat capacity (*C*_p_) of KCu_4_Se_3_ was measured and plotted as *C*_p_/*T* vs. *T*^2^ based on the Debye–Einstein model (Fig. 3b). The figure suggests that a low *C*_p_ results in low *κ*_l_ in KCu_4_Se_3_ (for more details, refer to Figs S7 and S8 and Table S2). Furthermore, *C*_p_/*T*^3^ was plotted vs. *T* (inset of Fig. 3b) to highlight the acoustic and low-energy optical phonons. The Debye model generally characterizes the long-wavelength acoustic phonons at very low temperatures, giving a *T*^3^ term in *C*_p_, while the Einstein model reflects the optical branches [26]. Moreover, the broad ‘boson-like hump’ observed at around 12 K indicates the presence of low-energy Einstein vibration, confirming the presence of the low-energy optical phonons.

In addition, to assess the *κ*_l_ value of KCu_4_S_3_ and KCu_4_Se_3_, Fig. 3c provides a *κ*_l_ map for bulk solids, including different systems for achieving low intrinsic *κ*_l_ at room temperature, such as the caged systems of clathrates and skutterudites with complex unit cells [27,28]; solids comprising heavy atoms with rattling modes [9,12]; Bi_2_Te_3_, which comprises heavy elements [29]; Cu_2_Se-based phonon liquids [22]; IV–VI, V_2_–VI_3_ and V materials employing resonant bonding scenarios [30], and defect mechanism-based systems [31]. The *κ*_l_ data of KCu_4_S_3_ and KCu_4_Se_3_ were plotted at 300 K and 573 K, respectively. KCu_4_S_3_ and KCu_4_Se_3_ are characterized by their light elements and relatively low *κ*_l_. The average atomic mass (*M*) values of KCu_4_S_3_ and KCu_4_Se_3_ are 48.7 and 66.2 amu, respectively, which are considerably smaller than those of caged systems, solids with rattling modes and materials comprising heavy elements. Moreover, KCu_4_Se_3_ exhibits the smallest mass contrast ratio of elements among these compounds (Fig. 3d). The measured *κ*_l_ of KCu_4_S_3_ and KCu_4_Se_3_ exhibits the relationship *κ*_l_ ∝1/*T*, indicating that the intrinsic Umklapp scattering process is the dominant scattering mechanism. Thus, lattice thermal transport calculations were conducted based on the three-phonon processes, and the calculated *κ*_l_ (*κ*_l_^cal^) values were considerably low (Fig. S9) owing to the tetragonal symmetry. These *κ*_l_^cal^ values indicate the anisotropy of $\kappa _{\mathrm{l}}^a\ $= $\kappa _{\mathrm{l}}^{b\ }$> $\kappa _{\mathrm{l}}^c$. The *κ*_l_^cal^ data of KCu_4_S_3_ at 300 K are *κ*_a_ = *κ*_b_ ≈ 0.575 W/(m·K) and *κ*_c_ ≈ 0.223 W/(m·K), whereas those of KCu_4_Se_3_ at 300 K are *κ*_a_ = *κ*_b_ ≈ 0.210 W/(m·K) and *κ*_c_ ≈ 0.111 W/(m·K).

The transport properties (both experimental and calculated) suggest an intrinsically low *κ*_l_ in KCu_4_S_3_ and KCu_4_Se_3_, indicating a large lattice anharmonicity. To further comprehend the lattice dynamics and thermal transport properties, the Grüneisen parameter (*γ*), which can characterize the lattice anharmonicity of a crystalline solid, was estimated based on the sound velocity using ultrasonic measurements (Table S3) [26]. For example, the *γ* values of KCu_4_Se_3_ in the para and perp directions are ∼1.97 and 1.90, respectively, which are competitive values because most low-*κ*_l_ solids exhibit *γ* values of 1–2 [3]. Moreover, the average sound velocity (*v*_a_) of these two solids is low; the *v*_a_ values of KCu_4_S_3_ in the para and perp directions are 2098 and 2208 m/s, respectively, and those of KCu_4_Se_3_ are 1755 and 1828 m/s, respectively, which are extremely low. The elastic properties were also estimated (Table S3) to confirm the bonding stiffness of KCu_4_S_3_ and KCu_4_Se_3_. For example, the Young's modulus and shear modulus of KCu_4_Se_3_ were calculated to be ∼15 and 13 GPa, respectively, which are very low values.

**Figure 4.**
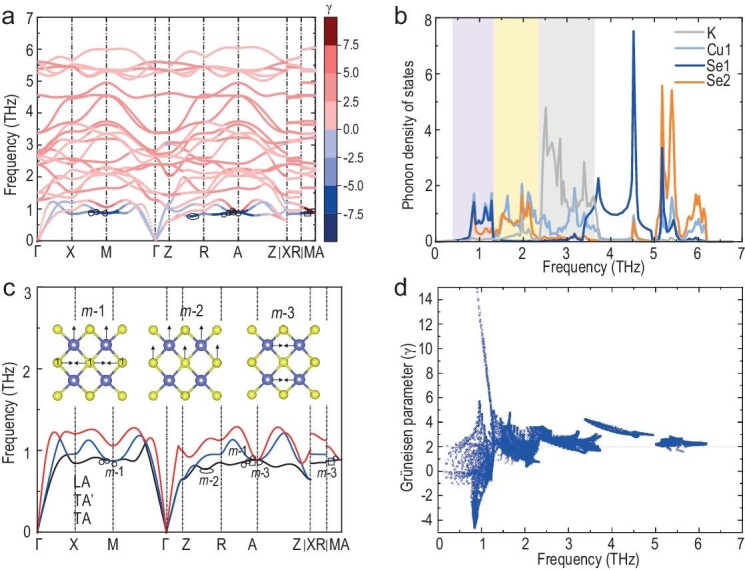
(a) Phonon dispersion and the color-coded mode Grüneisen parameters (*γ*) projected onto the phonon dispersion of KCu_4_Se_3_. (b) Phonon density of states of KCu_4_Se_3_. (c) Transverse (TA, TA’) and longitudinal (LA) acoustic phonon branches of KCu_4_Se_3_ (inset: phonon vibration modes in real space at the *κ* point, and energy indicated by circles for *m*-1, an ellipse for *m*-2 and squares for *m*-3.) (d) Frequency dependence of *γ* of KCu_4_Se_3_.

The phonon dynamics in KCu_4_S_3_ and KCu_4_Se_3_ were examined, and the results are illustrated in Fig. S10. Figure 4a depicts the phonon band dispersion of KCu_4_Se_3_, in which the phonon branches are quite flat, which is in good agreement with its low sound velocities. As shown in Fig. 4c, the transverse acoustic phonon modes (TA and TA’) and longitudinal acoustic phonon modes (LA) are labeled and exhibit a low cut-off frequency. The avoided crossing (∼1.0 THz) in the *Γ–Z* symmetry suggests an optical–acoustic coupling, indicating a strong phonon scattering process. This was confirmed by the color-coded mode *γ*, indicating its large phonon anharmonicity. The *γ* increases and is higher than 15 near the Brillouin zone center, reaching ∼5 at a frequency of ∼1.1 THz, which primarily originates from the Cu–Se^−^ and Cu–Se^2−^ modes (for more details, refer to Figs S10 and S11). The selected phonon modes (mode-1 (*m*-1), mode-2 (*m*-2) and mode-3 (*m*-3), indicated by a circle, an ellipse and a square, respectively) illustrate the active vibrations of Cu and Se^−^ at a low frequency and a large *γ*. These results are in good agreement with our PDF analysis, indicating that Cu and Se1(Se^−^) show large atomic displacement parameters. Furthermore, the phonon density of states (PhDOS) of different atomic species (Fig. 4b) indicates a considerable overlapping within a wide frequency range: (i) light K along with Cu are observed at high frequency (2.3–3.7 THz, gray shading); (ii) an overlap of the covalent Cu–Se^2−^ and ionic K–Se^2−^ bonding is observed at 1.3–2.3 THz (orange shading), which is confirmed by the similar PhDOS peaks of K, Cu and Se^2−^; (iii) a large contribution of Cu–Se^2−^ and Cu–Se^−^, of which the bigger contribution is from Cu–Se^−^, is observed at a low frequency (0–1.3 THz, blue shading).

**Figure 5.**
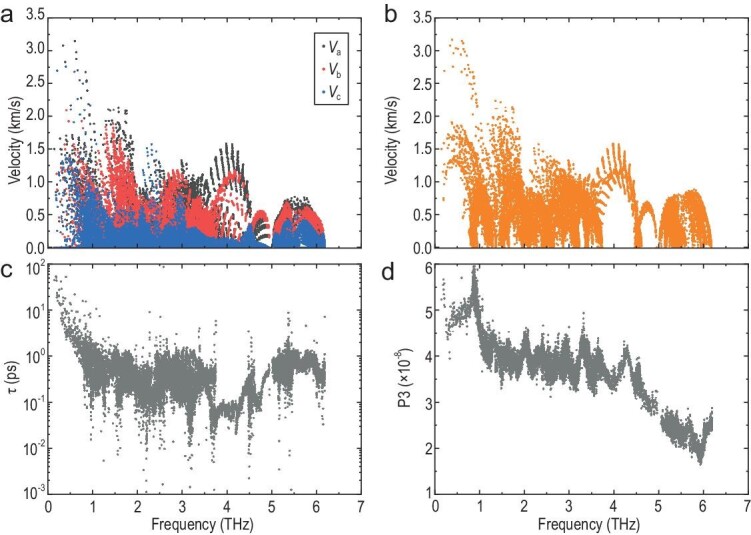
(a and b) Frequency dependence of the phonon group velocity and average group velocity of KCu_4_Se_3_. (c) Three-phonon relaxation time (τ). (d) Phase space for the three-phonon processes.

Despite its low atomic mass, KCu_4_Se_3_ has low group velocity (*v*) (Fig. 5a and b). The anisotropy of the phonon *v* is in good agreement with its tetragonal *P*4/*mmm* space group, which has acoustic modes with low *v* (<1.5 km/s) along the *z* direction. Moreover, all modes exhibit considerably low average *v* (<3.5 km/s) over most of the Brillouin zone (Fig. 5b). These group velocities are comparable to the experimental sound velocities, indicating a soft structural motif. Furthermore, the frequency-dependent relaxation time (*τ*) and phase space for the three-phonon processes (3 ph) of KCu_4_Se_3_ were determined in order to understand the detailed scattering mechanism (Fig. 5c and d). Large anharmonicity restricts the intrinsic *τ* in the time domain, and the low-frequency phonons show large dips in *τ* at frequencies corresponding to the avoided crossing, suggesting a strong anharmonic three-phonon scattering. Moreover, the large phase space for three-phonon processes indicates numerous available scattering channels in KCu_4_Se_3_, which can be attributed to its multiple phonon branch overlaps that satisfy the energy and momentum conservation requirement for 3 ph processes. In particular, the peak in the P3 curve of the phase space for three-phonon processes (Fig. 5d) at the frequency of the avoided crossing indicates the presence of a strong phonon scattering with acoustic–optical coupling. Thus, KCu_4_Se_3_ shows a strong anharmonic phonon scattering, involving extremely large phonon anharmonicity as well as large *γ* and phonon scattering channels, which are indicated by the large phase space for 3 ph processes. These phenomena collectively result in low *κ*_l_ in KCu_4_Se_3_.

To gain further insights into the low *κ*_l_ of KCu_4_Se_3_, which has a simple unit cell and light constituent elements, and the relationships between the structure and phonon transport, KCu_4_Se_3_ was compared to related compounds. For example, CsCu_4_Se_3_ [19] shares the same *P*4/*mmm* space group with KCu_4_Se_3_. Thus, it is expected that KCu_4_Se_3_, which contains lighter elements than CsCu_4_Se_3_, will exhibit lower *κ*_l_ than CsCu_4_Se_3_. However, the *κ*_l_ values of CsCu_4_Se_3_ are 0.48–0.20 W/(m·K) at 323 K–650 K [19], and those of KCu_4_Se_3_ are 0.43–0.12 and 0.86–0.21 W/(m·K) at 300 K–573 K in the para and perp directions, respectively. After excluding the experimental errors, the low *κ*_l_ of KCu_4_Se_3_ is confirmed. Therefore, we compared the crystal structures of the two compounds and found that KCu_4_Se_3_ depicts more asymmetric modules with a larger discrepancy in the [(Cu^+^)_4_(Se^2−^)_2_](Se^−^) sublattice (*d*_Cu–Se layer_ ≈ 5.85 Å) and [(K^+^)(Se^2−^)]^−^ sublattice (*d*_K–Se layer_ = 3.85 Å). In CsCu_4_Se_3_, the *d*_Cs–Se layer_ (4.43 Å) is larger than that of the *d*_K–Se layer_ owing to the larger radius of Cs, and the *d*_Cu–Se layer_ is ∼5.63 Å. Figure S11 shows a comparison between the phonon band dispersions of the two compounds. KCu_4_Se_3_ shows a frequency downshifting over the entire frequency range. The figure shows more dense phonon branches with barely considerable band gaps. In contrast to the sharp PhDOS peaks of Cs^+^ in CsCu_4_Se_3_, the PhDOS of K^+^ in KCu_4_Se_3_ shows large overlapping of the different atomic species of Cu and Se^2−^, indicating that their vibrations are greatly involved in the lower frequency range and suggesting the small discrepancy among the constituent atoms. This can strengthen the phonon dynamics in KCu_4_Se_3_, resulting in low *κ*_l_.

Another example is KCu_5_Se_3_ [32], which has the same constituent elements but exhibits a different crystallographic structure. KCu_5_Se_3_ crystallizes in the *P*4_2_/*mnm* space group (No. 136), and its complex unit cell (containing 36 atoms) is larger than that of KCu_4_Se_3_ (*N* = 8). Theoretically, KCu_5_Se_3_ should have lower *κ*_l_ than KCu_4_Se_3_ because of its complex lattice dynamics. However, its *κ*_l_ in the para direction is 0.58–0.49 W/(m·K) at 300 K–950 K, which is comparable, and even higher at high temperatures, to that of KCu_4_Se_3_. This may be attributed to the hierarchical structure, which contributes considerably to the low *κ*_l_ of KCu_4_Se_3_ compared with that of the complex KCu_5_Se_3_. These results indicate the effect of the crystal structure on the phonon dynamics and how they contribute to the low *κ*_l_ along with the light constituent elements with small discrepancy in atomic mass and radius, as well as the hierarchical structure in the simple square-net lattice.

## CONCLUSION

Two light-element solids, i.e. KCu_4_S_3_ and KCu_4_Se_3_, exhibit extremely low *κ*_l_ (0.15 and 0.12 W/(m·K), respectively) at 573 K, contradicting the conventional criteria that only heavy-element solids can have extremely low *κ*_l_. Herein, we confirmed that light constituent elements show small discrepancy in atomic mass and radius, along with a hierarchical structure in a simple square-net lattice, providing them with flat phonon branches and enhanced phonon interactions, which lead to a large phonon anharmonicity. The obtained low *κ*_l_ in light-element solids was lower than that of most heavy-element materials. The low *κ*_l_ of compounds composed of light elements is attributed to their square-net morphology, and it is predicted that if heavy elements are introduced into the square grid, lower *κ*_l_ values can be obtained. This phenomenon can also be used to overcome the *κ*_l_ limit in crystalline materials, allowing the suppression of thermal conduction in various materials. This work might reshape our fundamental understanding of the thermal transport properties of materials and advance the design of low-*κ*_l_ solids composed of light elements.

## METHODS

The experimental details are given in the supplementary data.

## Supplementary Material

nwae345_Supplemental_File
