# Supplementary material for: Extremely low lattice thermal conductivity in light-element solid materials
Source: Natl Sci Rev. 2024 Sep 28;12(1):nwae345. doi: 10.1093/nsr/nwae345 (PMC11702647; doi:10.1093/nsr/nwae345)
Supplement: nwae345_Supplemental_File [file nwae345_supplemental_file.zip › Supplemenatry data.pdf]

Extremely Low Lattice Thermal Conductivity in Light-element Material

Ni Ma[1†], Lu Liu[2†], Runhua Wu,[1] Juping Xu,[3] Wen Yin,[3] Kai Li,[1] Wei Bai,[1] Jiong Yang[2,4]*, Chong Xiao[1]*, Yi Xie[1]

[1]Hefei National Laboratory for Physical Sciences at the Microscale, University of Science and Technology of China, Hefei, Anhui, 230026, P. R. China.

[2]Materials Genome Institute, Shanghai University, Shanghai, 200444, P. R. China.;

[3]Spallation Neutron Source Science Center, Dongguan, 523803, P. R. China.;

[4]Zhejiang Laboratory, Hangzhou, Zhejiang 311100, P. R. China.;

EXPERIMENTAL SECTION

Synthesis and Characterization. Reagents of amorphous B powders (99.99%), K_2CO_3 powders (99.99%), Cu powders (99.999%), and Se powders (99.999%) were used as purchased from Alfa Aesar. The synthesis procedure was inspired by the boron-chalcogen method,[S1] plenty of shining KCu_4Se_3 crystals were obtained by melting K_2CO_3, Cu, B, and Se with stoichiometric ratio of K_2CO_3/Cu/B/Se = 3/24/2/18 at 973 K for 3 h then slowly cooled to room temperature. The room temperature powder X-ray diffraction (PXRD) measurement was performed on Rigaku TTR-III, with Cu Kα radiation λ = 1.5406 Å. The high-angle annular dark-field scanning transmission electron microscopic (HAADF-STEM) images were collected on a Titan Themis Z microscope equipped with probe and image correctors operated at 300 kV. Semi-quantitative elemental analysis was performed on a scanning electron microscope (SEM) (HITACHI SU-8220) equipped with an energy dispersive spectroscopy (EDS). The X-ray Photoelectron Spectroscopy (XPS) was collected on an ESCALAB 250Xi to interrogate the valence states of K, Cu, Se elements with base pressure of ~2×10^{-9} Torr.

Neutron Atomic Pair Distribution Function (PDF). Finely ground powder of KCu_4Se_3 was measured at 300 K, 373 K, and 473 K, respectively. The total neutron scattering data were processed using the Mantid software[S2] and Fourier transformation to obtain the pair distribution functions with $Q_{\min}$ = 0.5 Å^{-1}, $Q_{\max}$ = 31 Å^{-1}. The G(r) is the Fourier transform of the experimentally scattering structure function S(Q), G(r) = ($2/\pi$) $\int_{Q=0}^{Qmax} Q[S(Q)-1]Sin(Qr)dQ$,[S3] here, Q is the momentum transfer of the scattering particle. The PDF analysis was carried out using the PDFgui[S4] modeling platform.

Transport Property Measurement. Finely ground samples were consolidated by spark plasma sintering (SPS-211LX, Fuji Electronic Industrial Co., Ltd.) at 623 K for 10 min under an axial pressure of 40 MPa. The electrical conductivity σ and Seebeck coefficient S were measured on the commercial ZEM-3 (Ulvac Riko, Inc.). The total thermal conductivity (κ_{tot}) was calculated from the formula $\kappa = D \times C_p \times d$, where the thermal diffusivity (D) and specific heat (C_p) were obtained on a Netzsch LFA–457, the density (d) determined by the measured mass and dimensions. The densities of KCu_4S_3 and KCu_4Se_3 samples are given in Table S5. As shown in Figure S5 and Figure S6, the large κ_{tot} of KCu_4S_3 and KCu_4Se_3 are feasible, benefiting from its metallic conductivity with high σ value. The lattice thermal conductivity (κ_l) was obtained by subtracting electrical thermal conductivity ($\kappa_{ele}= L\sigma T$) from the κ_{tot}, where, Lorenz number $L \approx 1.5 + \exp\left[\frac{-|S|}{116}\right]$.[S5] The combined uncertainty for the transport property measurement is about 15%,[S6] thus, we marked the error bars for κ_l data in Figure 3a.

Low-temperature Heat Capacity. The low-temperature heat capacity of KCu_4Se_3 crystal (~6.1 mg) was measured on the PPMS by using APIEZON N grease in the temperature range of 2−200 K.

Ultrasonic Pulse Echo Measurement. The longitudinal (v_l) and transverse sound

velocities (v_t) were measured by using a commercial equipment (Ultrasonic Pulser/Receiver Model 5058 PR, Olympus, USA). The calculated average velocity v_a, Young's modulus E, shear modulus G, Poisson ratio v_ρ, and Grüneisen parameter γ are given in the Table S3, according to the following formulas:[S7–S9]

$$v_a = \left[\frac{1}{3}\left(\frac{1}{v_l^3} + \frac{2}{v_t^3}\right)\right]^{-1/3}, \text{(S1)}$$

$$E = \frac{\rho v_t^2(3v_l^2 - 4v_t^2)}{v_l^2 - v_t^2}, \text{(S2)}$$

$$v_\rho = \frac{1 - 2(\frac{v_t}{v_l})^2}{2 - 2(\frac{v_t}{v_l})^2}, \text{(S3)}$$

$$G = \frac{E}{2(1+v_\rho)}, \text{(S4)}$$

$$\gamma = \frac{3}{2}\left(\frac{1+v_\rho}{2-3v_\rho}\right), \text{(S5)}$$

Theoretical Calculations. Density functional theory (DFT)[S10,S11] calculations were performed using Vienna *ab* initio Simulation Package (VASP)[S12] based on PBE (Perdew-Burke-Ernzerhof) functional of generalized gradient approximation (GGA)[S13] with the projector augmented wave (PAW) method,[S14] and the kinetic energy cutoff of KCu_4Se_3 is 400 eV. Based on the Boltzmann transport equation, we use the Phonopy code,[S15] Thirdorder.py script[S16] and ShengBTE package[S17] with DFT package VASP to calculate the harmonic and anharmonic phonon scattering processes. The Brillouin-zone integrations used a Γ-centered k-point mesh of 16×16×7 during optimizing structure, and 1×1×1 for the phonon calculations. The convergence criteria for electronic and ionic relaxations were set as 5×10^{-8} eV and 5×10^{-5} eV/Å respectively for all DFT calculations. To simulate phonon with harmonic approximation and three-phonon scattering processes, 4×4×2 supercell structure was used in the DFT calculations. In lattice thermal transport calculations, 30×30×15 q-grid and 0.1 of scale parameter for Gaussian smearing were set based on the balance of accuracy and computing power.

FIGURES and TABLES.

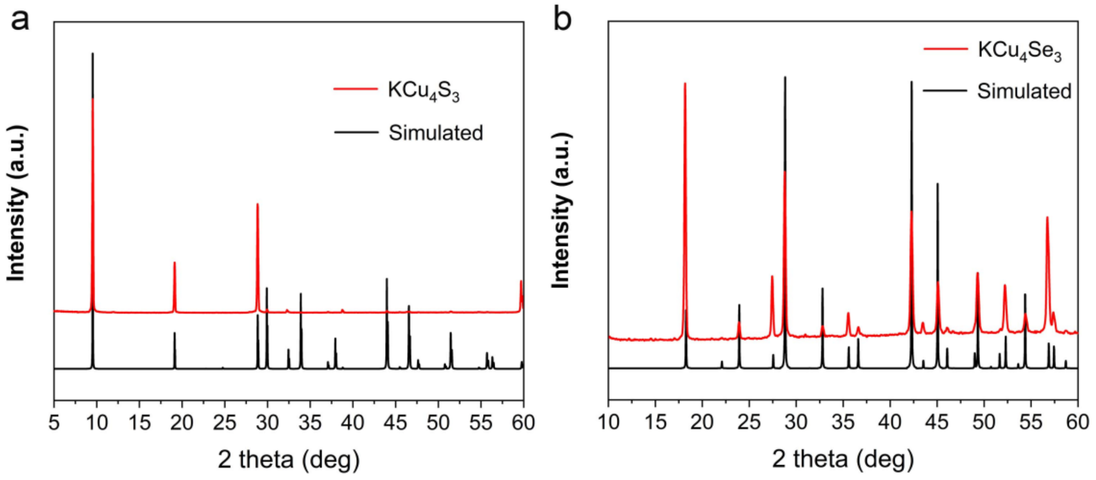

Figure S1. The PXRD patterns of the as-synthesized KCu_4S_3 and KCu_4Se_3.

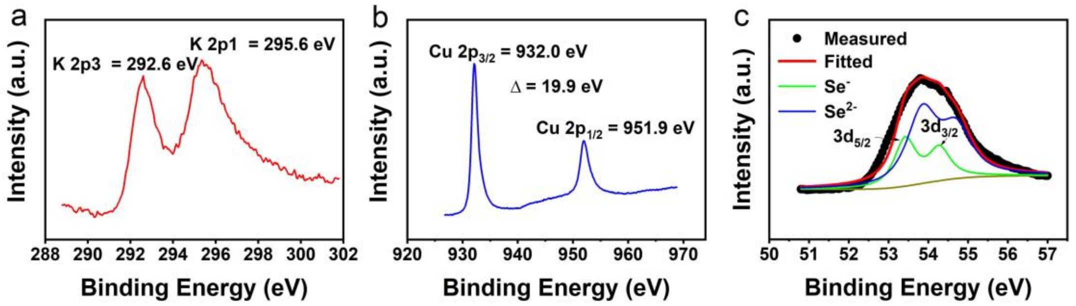

Figure S2. X-ray photoelectron spectroscopy of KCu_4Se_3. Spectra curve for K 2p, Cu 2p, and the Se 3d core levels, respectively. The K 2p core level is distinguished by the binding energies (BE) of 292.6 eV and 295.6 eV.[S18] The two peaks of 932.0 eV ($2p_{3/2}$) and 951.9 eV ($2p_{1/2}$) reveal the monovalent Cu^{+} with a separation of 19.9 eV.[S19] Two types of Se^{-} (Se1, Wyckoff site 1*d*) and Se^{2-} (Se2, Wyckoff site 2*g*) with BE of 53.42 eV ($3d_{5/2}$), 54.28 eV ($3d_{3/2}$), and 53.83 eV ($3d_{5/2}$), 54.69 eV ($3d_{3/2}$),[S1,S19] respectively. Additionally, the mixed valence states of chalcogen can be found in this class of copper chalcogenides, *i.e.*, $NaCu_4Se_4$,[S19] $NaCu_6Se_4$,[S20] and isostructural $CsCu_4Se_3$,[S1] in this mixed-valent systems, only Cu^{+} exists with the BE separation of ~19.9 eV that confirmed by XPS study, the mixed valence states mainly on the chalcogen that generate the delocalized electrons.

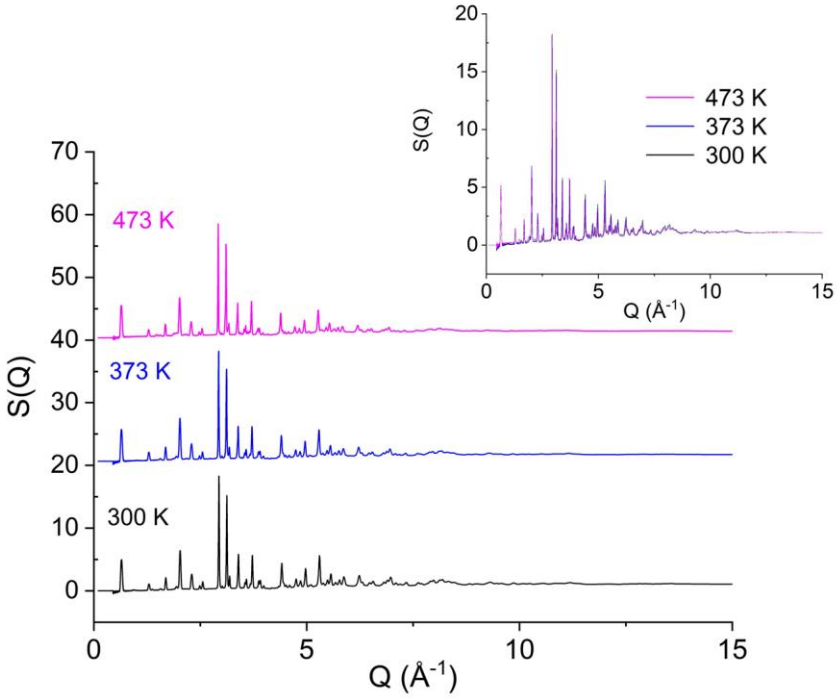

Figure S3. S(Q) vs Q for KCu_4Se_3 at 300 K (black), 373 K (blue) and 473 K (pink).

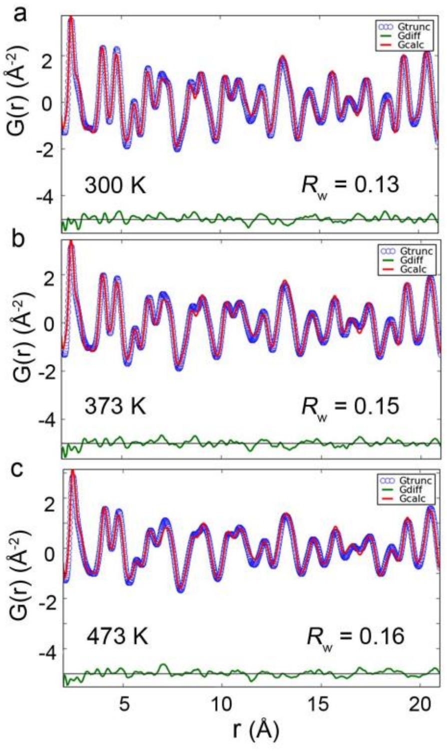

Figure S4. Neutron atomic pair distribution function (PDF) data of KCu_4Se_3 at 300 K (a), 373 K (b), and 473 K (c) fitted with tetragonal model with r = 2−20 Å.

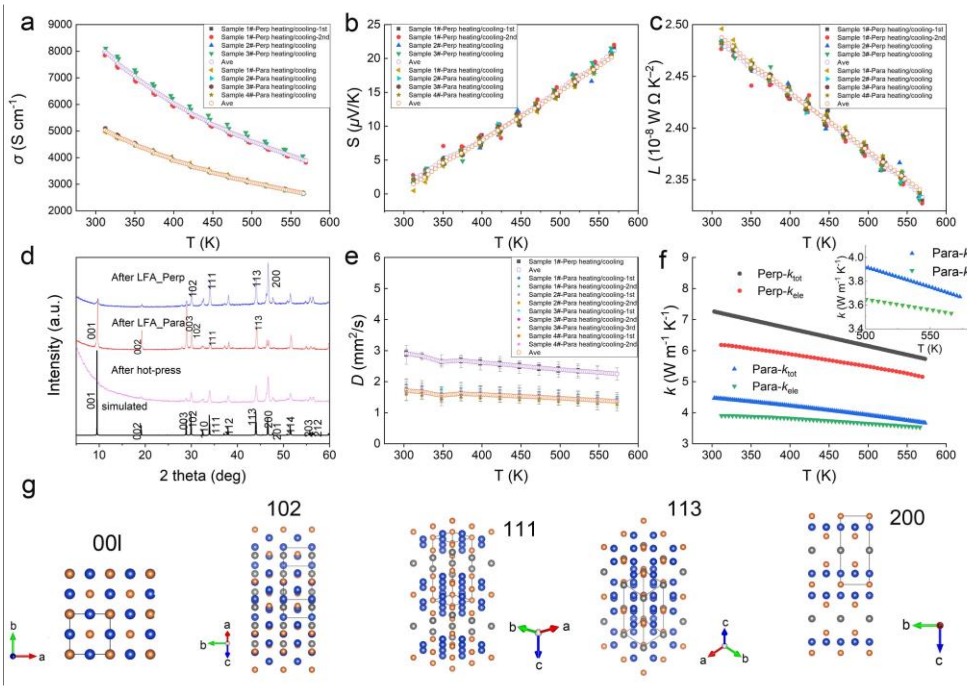

Figure S5. (a) Temperature-dependent electrical conductivity (***σ***), (b) Seebeck coefficient (***S***), (c) Lorenz number (***L***). (d) PXRD patterns of densified pellets along the perpendicular (perp) and parallel (para) directions. With the aid of PXRD results, we confirm the single-phase feature of KCu_4S_3 after SPS, before and after LFA measurement. (e) Temperature-dependent thermal diffusivities (***D***). (f) Total ($\boldsymbol{\kappa_{tot}}$) and electrical ($\boldsymbol{\kappa_{ele}}$) thermal conductivity of KCu_4S_3. Inset: the expand view of thermal conductivity in the range of 500−580 K of para samples. To verify the reproducibility of data, we performed the electrical properties (***σ***, ***S***) and thermal diffusivity (***D***) measurement on several samples, respectively. Heating and cooling data indicate the stability of transport properties. (g) Structures viewed along 00l, 102, 111, 113 and 200 of KCu_4S_3.

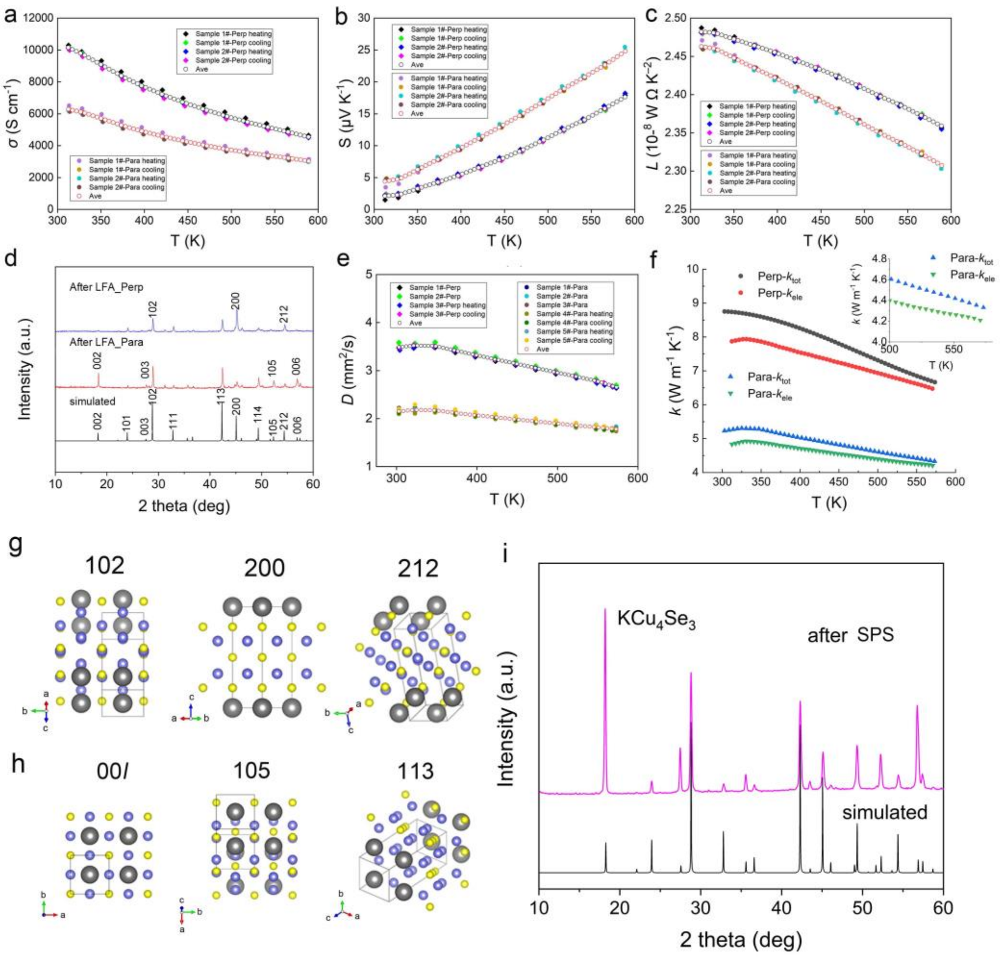

Figure S6. (a) Temperature-dependent electrical conductivity (***σ***), (b) Seebeck coefficient (***S***), (c) Lorenz number (***L***). (d) PXRD patterns of densified pellets along the perpendicular (perp) and parallel (para) directions with major diffraction indexes (*hkl*) marked. (e) Temperature-dependent thermal diffusivities (***D***). (f) Total (κ_{tot}) and electrical (κ_{ele}) thermal conductivity of KCu_4Se_3. Inset: the expand view of thermal conductivity in the range of 500−580 K of para samples. To verify the reproducibility of data, we performed the electrical properties (***σ***, ***S***) and thermal diffusivity (***D***) measurement on several samples, respectively. Heating and cooling data indicate the stability of transport properties. (g) Schematics of pressure directions, structures viewed along 102, 200 and 212 for perp sample, (h) 00*l*, 105 and 113 for para sample, indicating sample was roughly consolidated along *c* axis, which is consistent with the lower transport properties of para samples than that of perp samples. (i) PXRD of finely ground KCu_4Se_3 powder after SPS.

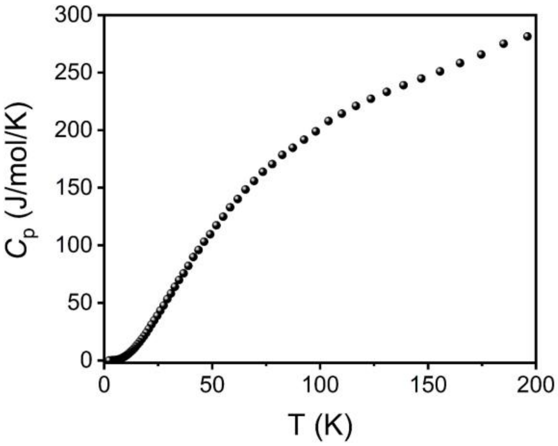

Figure S7. The curve of C_p vs T at low temperature.

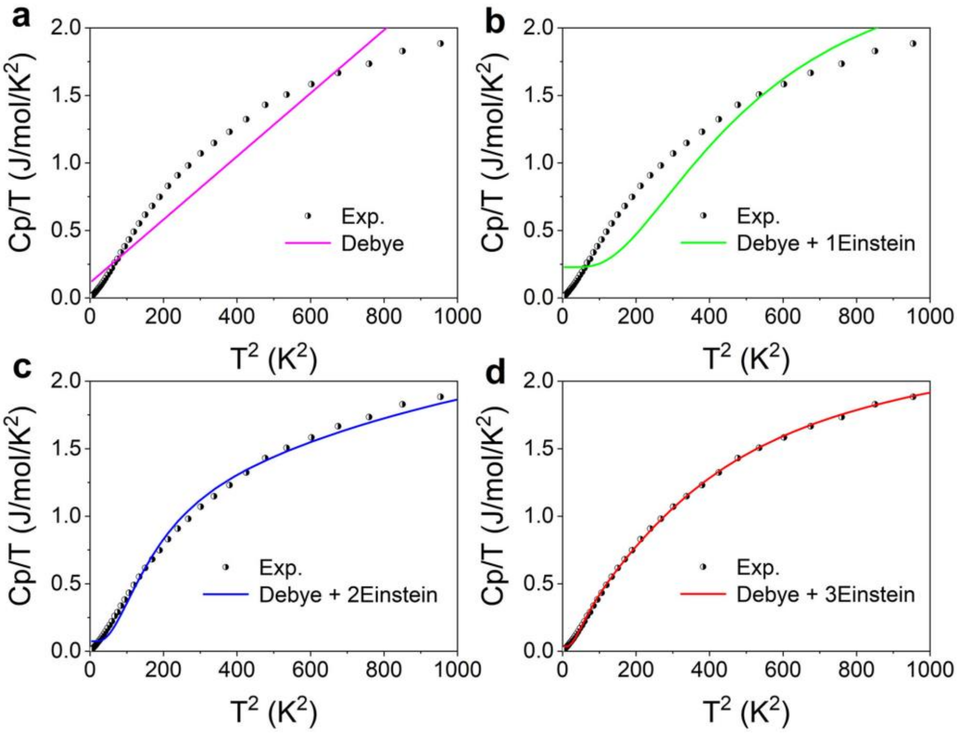

Figure S8. The C_p/T vs T^2 fitting plots according to Debye and multiple Einstein models. a) Debye model; b) Debye + 1 Einstein model; c) Debye + 2 Einstein model; d) Debye + 3 Einstein model. Black dots indicate the experimental heat capacity data.

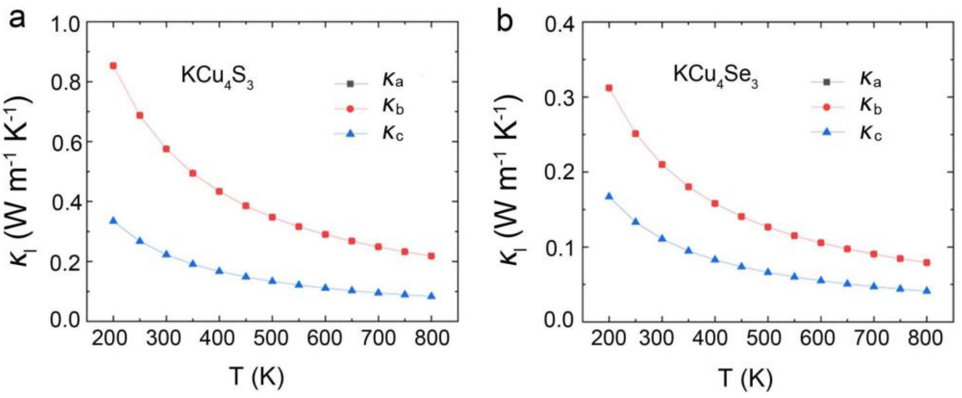

Figure S9. (a,b) Calculated lattice thermal conductivity κ_l of square-net KCu_4S_3 and KCu_4Se_3, respectively.

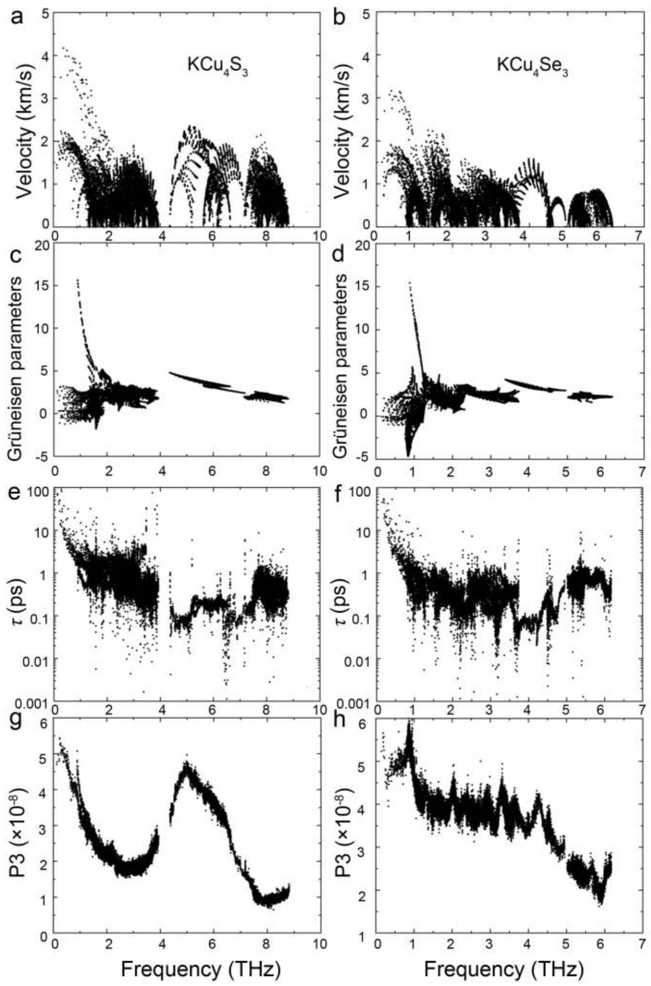

Figure S10. (a,b) Frequency-dependent group velocity, (c,d) Grüneisen parameters, (e,f) Three-phonon relaxation time (g,h) Phase space (P3) for square-net compounds KCu_4S_3 and KCu_4Se_3, respectively.

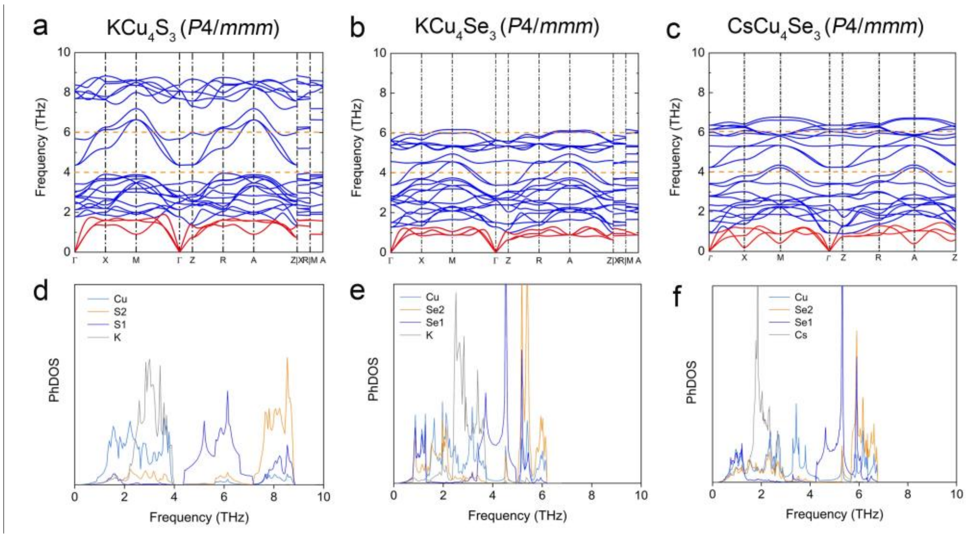

Figure S11. Comparison view of phonon dispersion (a−c) and PhDOS (d−f) of square-net KCu_4S_3, KCu_4Se_3 and $CsCu_4Se_3$[S1], all these compounds crystallize in space group *P*4/*mmm*. The atomic identity of each atom endows different building modules of these compounds, KCu_4Se_3 depicts asymmetric modules with smaller interlayer distance ratio of (KCu_4S_3; $d_{K\text{−}S2\ layer}/c$) vs (KCu_4Se_3; $d_{K\text{−}Se2\ layer}/c$) vs ($CsCu_4Se_3$; $d_{Cs\text{−}Se2\ layer}/c$): 0.41 vs 0.39 vs 0.44, such asymmetric modules of KCu_4Se_3 can be the chemical origin of the soft phonon modes and enhanced phonon-phonon interactions. Besides, phonon dispersions give more details, selenides show the evident frequency downshifting in comparison with sulfide, highlighted by orange dashed line in Figure S11a−c, suggesting the profound interaction of Cu and Se atoms. Regarding to the difference of alkali metals, we note that the middle-frequency of 2-4 THz has most of its contribution coming from K, instead of the Cs contributes most in the lower frequency <3 THz. KCu_4Se_3 shows strengthen synergy of various bonding of ionic K−Se^{2-}, covalent Cu−Se^{2-} and weaker Cu−Se^{-}, evidenced by the comparable contribution of each atom of PhDOS in the low frequency of 0−2.3 THz, thus, benefiting from the profound interaction of constituent light elements, KCu_4Se_3 still possesses a giant intrinsic anharmonicity that can realize low lattice thermal conductivity similar to those of heavy-element compounds.

Table S1. SEM and EDS spectrum of KCu_4S_3 and KCu_4Se_3 single crystal.

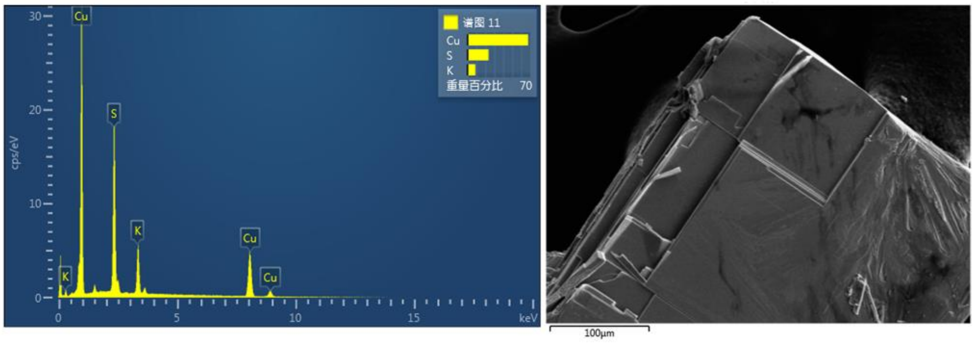

Element	K Ka Atomic %	Cu Ka Atomic %	S La Atomic %	Total Atomic %
Point 1	12.28	50.14	37.58	100
Point 2	12.35	50.12	37.53	100
Point 3	12.27	50.14	37.59	100
Average Formula		$KCu_{4.0(7)}S_{3.0(5)}$		

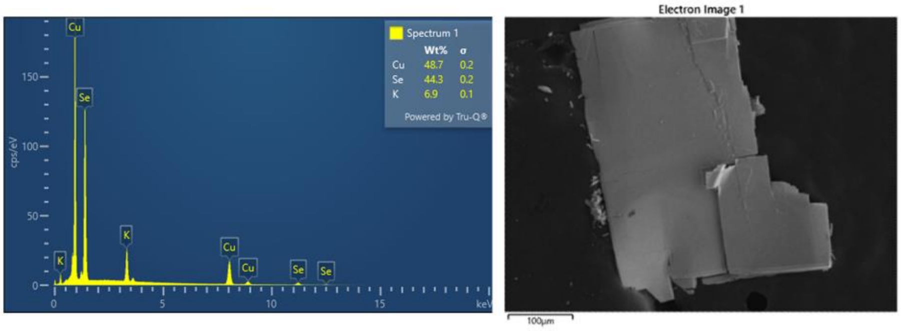

Element	K Ka Wt%	Cu Ka Wt%	Se La Wt%	Total Wt%
Point 1	11.74	50.96	37.30	100
Point 2	12.20	50.88	36.91	100
Point 3	12.21	50.50	37.28	100
Average Formula		$KCu_{4.2(1)}Se_{3.0(1)}$		

The C_p/T versus T^2 was fitted according to the Debye-Einstein model:[S21]

$$\frac{C_P}{T} = \gamma + \beta T^2 + \sum_i \left\{ A_i (\Theta_{Ei})^2 \cdot (T^2)^{-3/2} \cdot \frac{e^{\Theta_{Ei}/T}}{(e^{\Theta_{Ei}/T}-1)^2} \right\}, \text{(S6)}$$

where the Sommerfeld constant $\gamma = \frac{\pi^2}{3} k_B^2 N(E_F) = 1.36 \times 10^{-4} \times V_{mol}^{2/3} n_\gamma^{1/3} \frac{m^*}{m_0}$ is the electronic contribution to the heat capacity, the second term indicates the Debye lattice contribution of $\beta = B\cdot(12\pi^4 N_A \kappa_B/5)\cdot \Theta_D{}^{-3}$, and the parameter B can be assigned as $B = 1 - \sum_i A_i/3NR$ with R = 8.314 J/mol/K, and N is the number of atoms per formula unit. The last term is the Einstein oscillator modes, where A_i and Θ_{Ei} are the amplitude and the Einstein temperature of the ith Einstein oscillator mode, respectively, and Θ_D is the Debye temperature.

Table S2. Parameters obtained from fitting the experimental C_p/T-T^2 plot for KCu_4Se_3 when introducing different number of Einstein oscillators in the combined Debye−Einstein model.

**Parameter**	**Debye**	**Debye+1E**	**Debye+2E**	**Debye+3E**
γ/J mol^{-1} K^{-2}	0.1356	0.0434	0.0262	0.01574
β/J mol^{-1} K^{-4}	0.0024	0.001	0.0005	0.00031
$\mathbf{A_1}$/J mol^{-1} K^{-1}	/	42.08	65.38	1.87
$\boldsymbol{\Theta_{E1}}$/K	/	63.39	84.44	26.88 (~18.67 cm^{-1})
$\mathbf{A_2}$/J mol^{-1} K^{-1}	/	/	9.17	27.83
$\boldsymbol{\Theta_{E2}}$/K	/	/	39.81	60.17 (~41.81 cm^{-1})
$\mathbf{A_3}$/J mol^{-1} K^{-1}	/	/	/	80.41
$\boldsymbol{\Theta_{E3}}$/K	/	/	/	118.98 (82.68 cm^{-1})
$\boldsymbol{\Theta_D}$/K	/	/	/	141.02
$\mathbf{R^2}$	0.9019	0.9974	0.9996	0.99995
χ^2	2.31217	0.00107	0.00016	0.00002

Table S3. Room temperature elastic properties for KCu_4S_3 and KCu_4Se_3.

**Parameters**	**KCu_4S_3**		**KCu_4Se_3**	
	**para**	**perp**	**para**	**perp**
v_l (m s^{-1})	3461	3674	3072	3293
v_t (m s^{-1})	1881	1978	1567	1629
v_a (m s^{-1})	2098	2208	1755	1828
E (GPa)	41	46	36	39
G (GPa)	15	17	13	14
v_p	0.29	0.29	0.32	0.33
γ	1.71	1.71	1.97	1.90

Table S4. Sound velocities along different directions in the Brillouin zone.

**KCu_4S_3**			
**Parameters**	**Γ–X**	**M–Γ**	**Γ–Z**
v_{TA}**(m/s)**	1714	1900	1534
$v_{TA'}$**(m/s)**	1882	1825	1544
v_{LA}**(m/s)**	3013	3285	3201
**KCu_4Se_3**			
**Parameters**	**Γ–X**	**M–Γ**	**Γ–Z**
v_{TA}**(m/s)**	1116	1556	1364
$v_{TA'}$**(m/s)**	1592	1638	1332
v_{LA}**(m/s)**	2430	2405	2661

Table S5. Densities of KCu_4S_3 and KCu_4Se_3 samples.

KCu_4S_3	Expt. Density (g/cm^3)	Calc. Density (g/cm^3)	Relative Density (%)
Sample 1#-Perp	4.40	4.59	95.8
Sample 1#-Para	4.42	4.59	96.2
Sample 2#-Para	4.45	4.59	96.9
Sample 3#-Para	4.43	4.59	96.5
Sample 4#-Para	4.46	4.59	97.1
KCu_4Se_3	Expt. Density (g/cm^3)	Calc. Density (g/cm^3)	Relative Density (%)
Sample 1#-Perp	5.38	5.61	95.9
Sample 2#-Perp	5.40	5.61	96.2
Sample 3#-Perp	5.42	5.61	96.6
Sample 1#-Para	5.35	5.61	95.3
Sample 2#-Para	5.45	5.61	97.1
Sample 3#-Para	5.45	5.61	97.1
Sample 4#-Para	5.44	5.61	96.9
Sample 5#-Para	5.46	5.61	97.3

Reference

[S1] Ma, N.; Li, F.; Li, J. G.; Liu, X.; Zhang, D. B.; Li, Y. Y.; Chen, L.; Wu, L. M. Mixed-Valence $CsCu_4Se_3$: Large Phonon Anharmonicity Driven by the Hierarchy of the Rigid $[(Cu^+)_4(Se^{2-})_2](Se^-)$ Double Anti-CaF_2 Layer and the Soft Cs^+ Sublattice. *J. Am. Chem. Soc.* **2021**, *143*, 18490–18501.

[S2] Arnold, O. et al. Mantid-data Analysis and Visualization Package for Neutron Scattering and μSR Experiments. *Nucl. Instrum. Methods Phys. Res. A* **2014**, *764*, 156–166.

[S3] Egami, T. Billinge, S. Underneath the Bragg Peaks; Pergamon, **2012**.

[S4] Farrow, C. L. et al. PDFfit2 and PDFgui: Computer Programs for Studying Nanostructure in Crystals. *J. Phys.: Condens. Matter* **2007**, *19*, 335219.

[S5] Kim, H. S.; Gibbs, Z. M.; Tang, Y. L.; Wang, H.; Snyder, G. J. Characterization of Lorenz Number with Seebeck Coefficient Measurement. *APL Materials* **2015**, *3*, 041506.

[S6] Borup, K. A.; de Boor, J.; Wang, H.; Drymiotis, F.; Gascoin, F.; Shi, X.; Chen, L. D.; Fedorov, M. I.; Muller, E.; Iversen, B. B.; Snyder, G. J. Measuring Thermoelectric Transport Properties of Materials. *Energy Environ. Sci.* **2015**, *8*, 423−435.

[S7] Kurosaki, K.; Kosuga, A.; Muta, H.; Uno, M.; Yamanaka, S. Ag_9TlTe_5: A High-Performance Thermoelectric Bulk Material with Extremely Low Thermal Conductivity. *Appl. Phys. Lett.* **2005**, *87*, 061919.

[S8] Wan, C. L.; Pan, W.; Xu, Q.; Qin, Y. X.; Wang, J. D.; Qu, Z. X. Fang, M. H. Effect of Point Defects on the Thermal Transport Properties of $(La_xGd_{1-x})_2Zr_2O_7$: Experiment and Theoretical Model. *Phys. Rev. B* **2006**, *74*, 144109.

[S9] Tan, G.; Zhao, L. D.; Kanatzidis, M. G. Rationally Designing High-Performance Bulk Thermoelectric Materials. *Chem. Rev.* **2016**, *116*, 12123−12149.

[S10] Kohn, W. Nobel Lecture: Electronic Structure of Matter—Wave Functions and Density Functionals. *Rev. Mod. Phys.* **1998**, *71* (5), 1253−1266.

[S11] Jones, R. O.; Gunnarsson, O. The Density Functional Formalism, Its Applications and Prospects. *Rev. Mod.*

Phys. **1989**, *61* (3), 689−746.

[S12] Kresse, G.; Hafner, J. Ab Initio Molecular Dynamics for Liquid Metals *Phys. Rev. B* **1993**, *47*, 558−561.

[S13] Perdew, John P.; Burke, Kieron.; Ernzerhof, Matthias. Generalized Gradient Approximation Made Simple. *Phys. Rev. Lett.* **1996**, *77*, 3865−3868.

[S14] Kresse, G.; Joubert, D. From Ultrasoft Pseudopotentials to the Projector Augmented-Wave Method. *Phys. Rev. B* **1999**, *59*, 1758−1775.

[S15] Togo, Atsushi.; Tanaka, Isao. First Principles Phonon Calculations in Materials Science. *Scripta Materialia.* **2015**, *108*, 1−5.

[S16] Li, W.; Lindsay, L.; Broido, D. A.; Stewart, Derek. A.; Mingo, Natalio. Thermal Conductivity of Bulk and Nanowire $Mg_2Si_xSn_{1-x}$ Alloys from First Principles. *Phys. Rev. B* **2012**, *86*, 174307.

[S17] Li, W.; Carrete, Jesús.; Katcho, Nebil A.; Mingo, Natalio. ShengBTE: A Solver of the Boltzmann Transport Equation for Phonons. *Comput. Phys. Commun* **2014**, *185*, 1747−1758.

[S18] Moulder, John F. et al. Handbook of X-Ray Photoelectron Spectroscopy. **1992**.

[S19] Chen, H. J.; Rodrigues, J. B.; et al. High Hole Mobility and Nonsaturating Giant Magnetoresistance in the New 2D Metal $NaCu_4Se_4$ Synthesized by a Unique Pathway. *J. Am. Chem. Soc.* **2019**, *141*, 635–642.

[S20] Sturza, M.; Malliakas, C. D.; Bugaris, D. E.; Han, F.; Chung, D. Y.; Kanatzidis, M. G. $NaCu_6Se_4$: A Layered Compound with Mixed Valency and Metallic Properties. *Inorg. Chem.* **2014**, *53*, 12191–12198.

[S21] Xie, H. Y.; Su, X. L.; Zhang, X. M.; Hao, S. Q.; Bailey, T. P.; Stoumpos, C. C.; Douvalis, A. P.; Hu, X. B.; Wolverton, C.; Dravid, V. P.; Uher, C.; Tang, X. F.; Kanatzidis, M. G. Origin of Intrinsically Low Thermal Conductivity in Talnakhite $Cu_{17.6}Fe_{17.6}S_{32}$ Thermoelectric Material: Correlations between Lattice Dynamics and Thermal Transport. *J. Am. Chem. Soc.* **2019**, *141*, 10905−10914.
