# Supplementary material for: Extremely low lattice thermal conductivity in light-element solid materials
Source: Natl Sci Rev. 2024 Sep 28;12(1):nwae345. doi: 10.1093/nsr/nwae345 (PMC11702647; doi:10.1093/nsr/nwae345)
Supplement: nwae345_Supplemental_File [file nwae345_supplemental_file.zip › Teaser text.docx]

This work demonstrates two square-net chalcogenides, composed of relatively light elements with small atomic numbers, that exhibit extremely low lattice thermal conductivity.
